# Retraction: Interaction between Nbp35 and Cfd1 Proteins of Cytosolic Fe-S Cluster Assembly Reveals a Stable Complex Formation in *Entamoeba histolytica*

**DOI:** 10.1371/journal.pone.0281394

**Published:** 2023-01-30

**Authors:** The PLOS ONE Editors

Following the publication of this article [1], concerns were raised regarding results presented in Figs. 2, 6, 8, and 9. Specifically,

- In Fig. 2A, the plot representing Protein and the plot representing Side chain appear similar.
- The results presented in Fig. 6B and Fig. 6D appear to have been prepared using spliced blots.
- There appear to be irregularities in the background noise in lane 6 of the Fig. 8B Anti-Nbp35 panel.
- There appear to be irregularities in the Fig. 9 panels:
  - In the (IgG) Cfd1 panel, around the 28kDa band in the lane marked as 1.
  - In the (IgG) Cfd1 panel, in the background between lanes marked as 4 and 5.
  - In the (IgG) Nbp35 panel around the 36kDa bands in the lanes marked as 1 and 2.

The corresponding author confirms that the results presented in Figs. 6B and 6D originate from spliced gels. As the original data have been obtained from separate gels, the marker lanes are not cross-comparable, and separate marker lanes should have been presented for each blot. The authors provided the underlying data and replicate experiment data for the lanes marked as 12 and 13 in Figs. 6B and 6D, but the original blots used to prepare the lanes marked as M and 11 in Figs. 6B and 6D have not been provided for editorial review.

The corresponding author stated that the original uncropped blot underlying the Fig. 8B results are no longer available. In the absence of the underlying data, the concerns with Fig. 8B cannot be resolved.

The original data underlying the Fig. 9 (IgG) Cfd1 results show that lane 5 contains a band that does not appear to be present in the published figure. The corresponding author clarified that the band of the heavy Ig-G chain in lane 5 may have been edited during the preparation of the figure, but stated that this does not alter the outcome of the immune-precipitation result. The editing of this band suggests this figure has not been prepared in line with the *PLOS ONE* figure preparation guidelines [2] in place at the time of the article’s submission.

In addition, the assessment of the underlying data provided for the Fig. 9 (IgG) Cfd1 results showed that the band presented at the 28kDa marker in the lane marked as 1 does not line up with the bands presented at the 28kDa marker in the lanes marked as 2 and 3. Instead, in the underlying data the band in the lane marked as 1 is estimated at approximately 30kDa in the underlying data.

The original data underlying the Fig. 9 (IgG) Nbp35 blot have not been provided for editorial review. In the absence of the underlying data, the concerns with the Fig. 9 (IgG) Nbp35 blot cannot be resolved.

The underlying data for Fig. 2A have been provided for editorial review, but in light of other concerns raised with this article, these underlying data have not been fully investigated.

The authors provided underlying data for Figs. 2B, 6B, 6D, 8A, 8B, and 9 (Cfd1 panel only). The underlying data used to prepare the results presented in the remaining figures either is no longer available or has not been provided for editorial review.

In light of the concerns affecting the results presented in Fig. 8B and Fig. 9 that question the integrity of these data, the *PLOS ONE* Editors retract this article.

VA did not agree with the retraction. SA, MRD, KPS, RKK, AZ, GCS, AKR, TN, and PD either did not respond directly or could not be reached.
